# P-Type ZnO Films Made by Atomic Layer Deposition and Ion Implantation

**DOI:** 10.3390/nano14131069

**Published:** 2024-06-22

**Authors:** Guoxiu Zhang, Lars Rebohle, Fabian Ganss, Wojciech Dawidowski, Elzbieta Guziewicz, Jung-Hyuk Koh, Manfred Helm, Shengqiang Zhou, Yufei Liu, Slawomir Prucnal

**Affiliations:** 1 Helmholtz-Zentrum Dresden-Rossendorf, Institute of Ion Beam Physics and Materials Research, Bautzner Landstrasse 400, 01328 Dresden, Germany; 2 Key Laboratory of Optoelectronic Technology & Systems, Ministry of Education, Chongqing University, Chongqing 400044, China; 3 Helmholtz-Innovation Laboratory Blitzlab, Helmholtz-Zentrum Dresden-Rossendorf, Bautzner Landstrasse 400, 01328 Dresden, Germany; 4 Faculty of Electronics, Photonics and Microsystems, Wrocław University of Science and Technology, Janiszewskiego 11/17, 50-372 Wrocław, Poland; 5 Institute of Physics, Polish Academy of Sciences, Al. Lotników 32/46, 02-668 Warsaw, Poland; 6 Department of Intelligent Energy and Industry, Chung-Ang University, Seoul 06974, Republic of Korea; 7 Institute of Applied Physics, Technische Universität Dresden, 01062 Dresden, Germany; 8 Faculty of Science and Engineering, Bay Campus, Swansea University, Swansea SA1 8EN, UK

**Keywords:** p-type-doped ZnO, atomic layer deposition, ion implantation, flash lamp annealing, rapid thermal annealing

## Abstract

Zinc oxide (ZnO) is a wide bandgap semiconductor that holds significant potential for various applications. However, most of the native point defects in ZnO like Zn interstitials typically cause an n-type conductivity. Consequently, achieving p-type doping in ZnO is challenging but crucial for comprehensive applications in the field of optoelectronics. In this work, we investigated the electrical and optical properties of ex situ doped p-type ZnO films. The p-type conductivity has been realized by ion implantation of group V elements followed by rapid thermal annealing (RTA) for 60 s or flash lamp annealing (FLA) on the millisecond time scale in nitrogen or oxygen ambience. The phosphorus (P)-doped ZnO films exhibit stable p-type doping with a hole concentration in the range of 10^14^ to 10^18^ cm^−3^, while antimony (Sb) implantation produces only n-type layers independently of the annealing procedure. Microstructural studies of Sb-doped ZnO show the formation of metallic clusters after ms range annealing and SbZn-oxides after RTA.

## 1. Introduction

Zinc oxide (ZnO), as a natural substance easily obtained through the calcination of lead–zinc ores, has been historically utilized for various applications. In its early application, low-purity ZnO was valued for its whiteness and UV resistance in the fields of coatings and pigments [1,2]. Subsequently, with the advancement of the chemical industry, ZnO found applications as a chemical additive in cement [3], ceramics [4], cosmetics, etc. As refining techniques improved, high-purity ZnO revealed further applications in the medical field due to its antimicrobial capabilities and biocompatibility [5,6]. It has been employed in topical pharmaceuticals, gastric antacid formulations, and biosensors, among other medicinal applications. With the arrival of the information technology age, ZnO as a semiconductor has emerged with considerable potential, attributed to its notably wide direct bandgap of about 3.37 eV and the remarkably large exciton binding energy of 60 meV [7,8]. In recent years, it has attracted more and more attention in many fields such as optoelectronic devices [9,10], industrial catalysis [11,12], biomaterials [5,6], and light emitters [13,14,15] due to its controllable optoelectronic properties, low cost, and non-toxicity.

Typically, not intentionally doped ZnO exhibits n-type conductivity as a result of point defects like zinc interstitials (Zn_i_) and oxygen vacancies (V_O_); thus, achieving n-type doping in ZnO is highly facile based on the background carriers [16,17]. Numerous studies have consistently reported electron concentrations in ZnO reaching up to ~10^21^ cm^−3^ [18,19], which substantiates the great potential of ZnO in optoelectronics and plasmonics. To fully exploit the unique properties of ZnO, controlled doping must be achieved for both n-type and p-type conductivity. However, due to the wide bandgap and low valence band energy, it is still challenging to obtain p-type ZnO [20,21,22]. Initially, the strategies to realize p-type doping of ZnO can be categorized into two main types: (1) replacing Zn^2+^ by utilizing positively charged ions such as lithium (Li) [23] or sodium (Na) [24,25] from group IA [26]; or (2) substituting O^2-^ by group VA elements such as nitrogen (N) and phosphorus (P) [27]. Another method that can produce p-type conductivity in ZnO is co-doping with two different elements, e.g., Ga and N, where Ga increases the solubility of N in ZnO and helps to achieve p-type doping through the formation of Ga-N acceptor levels [28,29]. A similar effect was achieved for Al and N co-doping of ZnO [30]. Moreover, the doping strategy can be distinguished between in situ and ex situ doping. The doping during the growth (in situ) allows for the fabrication of high-quality vertical devices both with the p-type and n-type conductivity [31]. In contrast, using ex situ doping, lateral devices on wafers with different functionalities can be made. The most common and precise ex situ doping method for modification of optoelectronic properties of semiconductors is ion implantation [32] which allows for the control of both the carrier concentration and the spatial distribution of dopants and doping much above the solid solubility limit. Unfortunately, the incorporation of dopants into the solid by ion implantation is accompanied by ion-induced crystal lattice degradation. Therefore, ion implantation doping is typically used together with post-implantation annealing. One of the most popular acceptors of ZnO is nitrogen. It was shown that ZnO implanted with nitrogen and subsequently annealed possesses a hole concentration of about 10^17^ cm^−3^ [33]. As was shown by first-principle calculations, nitrogen implanted in ZnO and incorporated into an oxygen sublattice (N_O_) is a deep acceptor (activation energy about 1.3 eV) and can be responsible for p-type conductivity [34]. N_O_ surrounded by Zn_i_, V_O_, and O_i_ forms a shallow donor level increasing the n-type conductivity of the ZnO layer [35]. Only N_O_ bonded with V_Zn_ may form stable p-type doping [36]. Similar to nitrogen, P substituting oxygen is a deep acceptor while substituting Zn forms a complex defect center P_Zn_–2V_Zn_ with an acceptor state about 150 meV above the valence band [37]. Sb-doped ZnO and annealed in nitrogen typically shows n-type conductivity with electron concentration above 10^20^ cm^−3^ but annealing in air produces p-type conductivity. To the best of our knowledge, the ZnO implanted with different group V elements has been subject to conventional thermal annealing only for those that may cause the diffusion of implanted elements, and the maximum carrier concentration is limited by the solid solubility of dopants in ZnO [38]. Moreover, the p-type poly-crystalline ZnO doped with group V elements by room temperature ion implantation has not been achieved so far. Therefore, for a better understanding of ion–solid interactions and the defect evolution in implanted ZnO layers subject to post-implantation annealing, there needs to be a more systematic investigation.

In this work, we present p-type poly-crystalline ZnO films fabricated by atomic layer deposition (ALD) and ion implantation of phosphorus followed by high-temperature annealing either in an oxygen or nitrogen ambient using both conventional rapid thermal annealing (RTA) for 60 s or advanced non-equilibrium thermal processing, i.e., millisecond (ms) range flash lamp annealing (FLA). The main advantage of FLA over other thermal processes is that within a few milliseconds, ion-implanted dopants are activated, and ion-induced defects are removed. Moreover, using FLA, doping is possible much above the equilibrium solid solubility [39]. The P-implanted samples, after FLA for 23 ms, show a hole concentration close to 2 × 10^18^ cm^−3^, while RTA-treated samples show a maximum hole concentration in the range of 10^16^ cm^−3^. The structural investigation reveals that after RTA the implanted Sb slightly diffuses out from the sample while FLA causes the Sb metallic cluster formation, and independently of the annealing method and ambient, the Sb-doped samples are n-type. Moreover, the annealing atmosphere affects mainly the optical properties of annealed layers due to the change in the point defect nature.

## 2. Experiment

**Figure 1:**
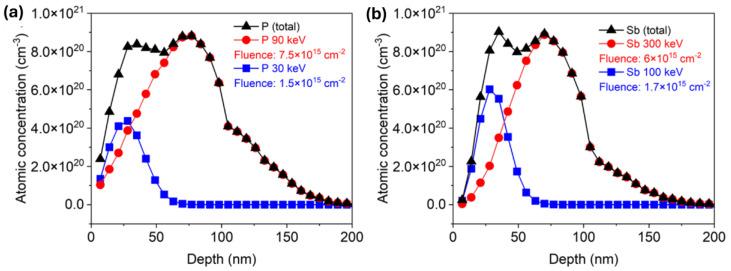
SRIM simulation of depth distribution of (**a**) phosphorus and (**b**) antimony in 100 nm ZnO film with high dose, respectively.

Around 100 nm thick ZnO films were deposited via atomic layer deposition (ALD) on Si wafers with a 100 nm thick SiO_2_ layer via Savannah 300C, either at 130 °C or 200 °C with a growth rate of 1.8 Å/cycle and 1.4 Å/cycle, respectively. Diethylzinc (DEZn) from Sigma-Aldrich (product no. 256781) was employed as a zinc precursor, and deionized water (H_2_O) was used as an oxidant for the ZnO layers. A continuous N_2_ flow of 0.3 Torr was applied as a carrier gas to introduce the precursors into the reaction chamber and employed as a purging gas to remove unwanted byproducts from the chamber at the same time. Both ZnO films were deposited with 25 ms pulsing time, while the purging time was 8 s and 16 s for DEZn and water, respectively. The deposited ZnO films are polycrystalline. More details about growing conditions can be found in the reference [22]. Next, ion implantation was applied to introduce phosphorus (P) or antimony (Sb) into the ZnO layer. The films were doped with two different dopant concentrations by a low and a high implantation dose. Specific implantation parameters are listed in Table 1. In order to ensure a homogeneous doping profile, two implantation energies were combined. Both energies were calculated individually for P and Sb using the SRIM code [40]. The calculated depth distributions for P and Sb after ion implantation with two energies are presented in Figure 1a,b, respectively. For phosphorus-doped ZnO (PZO) samples with a low dose, the first implantation was made at an energy of 90 keV with an implantation fluence of 7.5 × 10^15^ cm^−2^. The subsequent implantation was made at an ion energy of 30 keV and a fluence of 1.5 × 10^15^ cm^−2^. Due to the larger atomic mass of antimony compared to phosphorus, higher energy is necessary to ensure the same dopant distribution in ZnO. Sb^+^ ions were implanted into the ZnO film (SZO) first with an energy of 300 keV and an implantation fluence of 6 × 10^15^ cm^−2^ and next with an energy of 100 keV and a fluence of 1.7 × 10^15^ cm^−2^. In the case of high-dose samples, ion fluences for each implantation step were doubled. Within the content of this paper, samples implanted with lower and higher doses are indicated as LD and HD, respectively. The simulated depth distributions of implanted elements are shown in Figure 1. After ALD and ion implantation, the wafers were cut into 1.2 × 1.2 cm^2^ pieces and post-processed separately by RTA and FLA using different atmospheres. In the case of RTA, the samples were annealed at 800 °C for 60 s, while the pulse length of FLA was 23 ms. Both annealing methods were conducted in a continuous flow of N_2_ or O_2_.

To investigate the electrical properties of implanted and annealed samples, Hall effect measurements were made using the four-probe method in a Van der Pauw configuration (Ecopia HMS-3000 Hall Measurement System from Bridge Technology, Washington, D.C., USA) at room temperature (RT). The ohmic contact on the doped ZnO samples was made using a magnetron sputter deposition from Rovak through a shadow mask of a 20 nm thick Ni and an 80 nm thick Au layer. The circular contacts have a diameter of about 2 mm and Ni/Au should provide low-resistance ohmic contacts to p-type ZnO [41]. Photoluminescence (PL) spectra were measured using a 320 nm Nd-YAG laser (CW ultraviolet DPSS laser from Changchun New Industries Optoelectronics Technology Co., Ltd., Changchun 130103, P.R.China) with a power of 20 mW and a 340 nm long pass edge filter. Micro-Raman spectroscopy with a 532 nm Nd-YAG laser from Coherence and a liquid-nitrogen-cooled Si-CCD was used to measure the spectra in the wavenumber range between 50 and 900 cm^−1^. A D8 X-ray diffractometer (XRD) from Bruker, Karlsruhe, Germany, with a Cu Kα source was used to investigate the structural properties of the obtained films. The Bragg–Brentano scans were performed for diffraction angles ranging from 20° to 60°. Rutherford backscattering spectrometry random scans (RBS/R) were used to determine the element distribution of the SZO thin films treated by various thermal methods. RBS was performed at the Ion Beam Center of the Helmholtz-Zentrum Dresden-Rossendorf (HZDR), Germany, using a 2 MV van de Graaf accelerator. The energy of the He^+^ beam was 1.7 MeV, and the beam diameter was about 1 mm. The distribution of P in ZnO could not be detected by RBS due to the limited system resolution for elements lighter than the matrix elements.

## 3. Results and Discussion

### 3.1. Hall Effect Measurements

Table 2 shows the summary of the carrier type, concentration, and carrier mobility in PZO and SZO samples annealed under different conditions. The electrical characteristics of the films were determined through Hall effect measurements conducted at room temperature using the Van der Pauw configuration. Positive and negative numbers are for holes and electrons, respectively. The as-grown samples are n-type with an electron concentration in the range of 5–8 × 10^18^ cm^−3^. The as-implanted samples are n-type with an electron concentration above 10^20^ cm^−3^ for P-implanted ZnO and in the order of 5 × 10^19^ cm^−3^ after Sb implantation. In general, samples grown at higher temperatures or implanted with higher fluence exhibit higher electron concentrations. The ion implantation causes a significant lattice distortion and in extreme cases can lead to full amorphization of the crystal host. We assume that at the as-implanted stage, most of the implanted atoms are not incorporated into the crystal lattice and n-type conductivity is due to point defects caused by ion irradiation [42]. After annealing, samples recrystallize and at least a part of the implanted atoms are expected to be incorporated at the oxygen site in the crystal providing holes to the layer. In fact, after FLA, some of the P-doped samples show p-type conductivity with a hole concentration varying from 2.84 × 10^17^ cm^−3^ to 1.91 × 10^18^ cm^−3^, while all the samples treated by RTA show p-type conductivity with a hole concentration ranging from 1.55 × 10^14^ cm^−3^ to 3.38 × 10^16^ cm^−3^. The hole mobility for flash-annealed samples is in the range of 4 to 6 cm^2^/Vs, while, after RTA, it can reach 12 cm^2^/Vs in samples with a hole concentration of 1.87 × 10^16^ cm^−3^. Higher Hall mobility in samples annealed by RTA is mainly due to a lower doping level, i.e., lower Coulomb scattering. Sb-doped ZnO samples show either n-type conductivity after FLA or are too resistive to be measured after RTA. Antimony in ZnO is a deep acceptor with activation energy between 140 and 220 meV, depending on the doping level and location of Sb in the crystal [43]. Therefore at room temperature similar to Mg in GaN [44], it is difficult to estimate the active carrier concentration in moderately doped samples. The highly resistive layers achieved after RTA suggest that some of the Sb atoms are electrically active, but the holes are localized within the impurity band. To shine more light on the activation of acceptor states, we have performed structural and optical investigations of implanted and annealed samples.

### 3.2. Rutherford Backscattering Spectrometry

**Figure 2:**
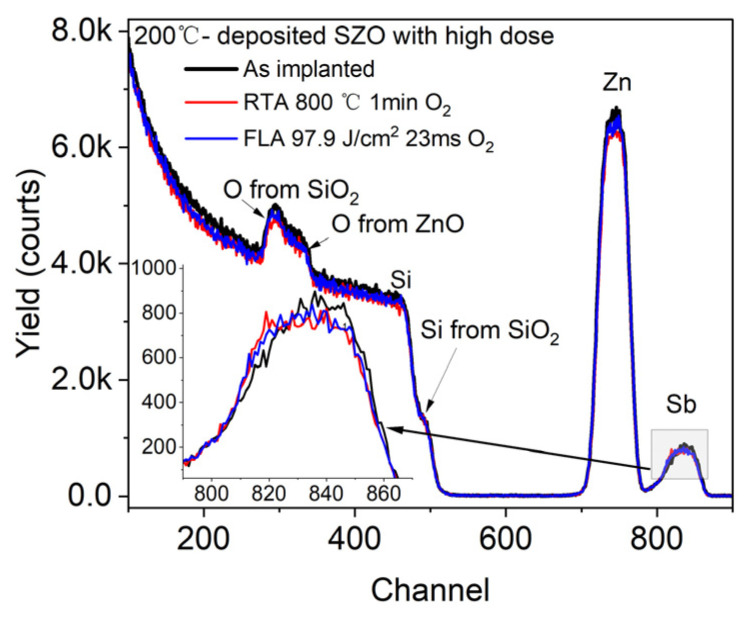
RBS/R spectra of ZnO films grown by ALD at 200 °C and implanted with a high dose of Sb. After ion implantation samples were treated via different annealing methods. The inset shows a magnified signal from Sb.

First, we verified the depth distribution of Sb in ZnO using RBS spectrometry. Figure 2 shows the RBS spectra of the ZnO layer grown at 200 °C and Sb^+^-implanted with a high dose before and after annealing. The sample with a high dose was chosen to increase the visibility of the dopant. Each element of the chemical composition in the films is recorded at different channels, which allows us to investigate the influence of applied annealing on the elemental depth distribution.

We can see that the ZnO peak has the same width as that of Sb, which means successful homogeneous doping of ZnO thin films with Sb impurities, which is consistent with the dopant distribution profile given by SRIM code simulation (see Figure 1). Importantly, there is almost no change in the Sb distribution after annealing. This means that both annealing conditions, FLA for 23 ms and RTA for 60 s, do not lead to a significant Sb diffusion. The Si signal from the Si substrate and from the SiO_2_ layer has a sharp edge and does not change after annealing. This suggests no diffusion between ZnO and SiO_2_ layers. Similar results are expected for P-doped ZnO. Unfortunately, the depth distribution of P in ZnO could not be confirmed by RBS due to the limited sensitivity to the elements lighter than the matrix.

### 3.3. Raman Spectroscopy

**Figure 3:**
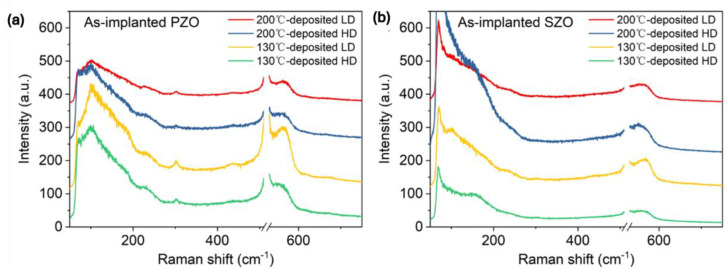
Raman spectra obtained from as-implanted ZnO films with (**a**) P ions and (**b**) Sb ions.

Next, we investigated the phonon spectra of the implanted and annealed ZnO. In the as-implanted stage, the ZnO layer is strongly disordered but not fully amorphous. ZnO is known to be very resistant to ion irradiation [45,46]. High fluence ion implantation with middle-range energies mainly causes some atom displacement and formation of various disorders in the crystal, like basal dislocation loops within the dopant distribution and prismatic loops located beyond the ion range [46]. Argon ions with an energy of 300 keV and an ion fluence of 4 × 10^16^ cm^−2^ are not enough to amorphize ZnO thin films [46]. Hence, we assume that our ZnO films after ion implantation with a high fluence of about 2 × 10^16^ cm^−2^ cannot be fully amorphized as well and only some crystal disorder is expected. Figure 3 shows Raman spectra recorded from as-implanted ZnO films with P and Sb ions at different ion doses. The phonon mode associated with the longitudinal optical mode in the Si substrate at 520 cm^−1^ has been subtracted from the spectra. The crystalline ZnO has two main Raman active phonon modes A_1_(LO) located at about 570 cm^−1^ and an E_1_ phonon mode at about 585 cm^−1^ [47].

In our case, all as-implanted samples show a broad peak at about 580 cm^−1^, possibly due to the broken symmetry, e.g., due to ion-induced defects, that can be assigned to the A_1_^(LO)^ mode superimposed with the E_1_ phonon mode [48,49]. In wurtzite crystal ZnO, the optical phonons, denoted as Γ_opt_, are expressed as the sum of vibrational modes: Γ_opt_ = 1A_1_ + 2B_1_ + 1E_1_ + 2E_2_. Wherein, A_1_ and E_1_ modes exhibit polarity and further divide into a transverse optical mode (TO) and a longitudinal optical mode (LO) (A_1_^(TO)^, A_1_^(LO)^, E_1_^(TO)^, E_1_^(LO)^) and they are all Raman active, while B_1_ modes behave as a silence mode. E_2_ modes are also Raman active, but they are nonpolar. After annealing, the implanted layers are crystalline and the ZnO is transparent for the green light used here. Therefore, the intensity of the Raman peaks after annealing is weaker. Nevertheless, Raman spectroscopy provides basic information about the recrystallization process of the implanted and annealed layer and the possible formation of clusters from the implanted elements.

**Figure 4:**
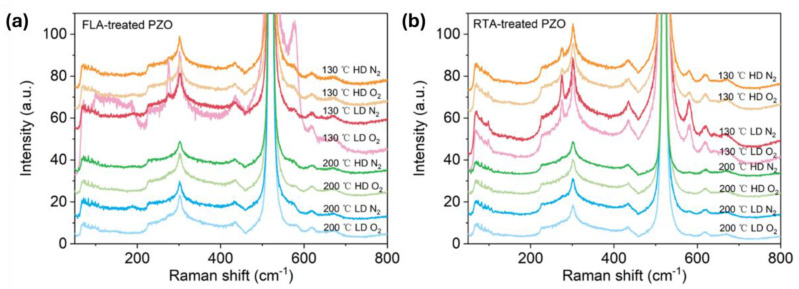
Raman spectra of P-implanted and annealed samples treated with FLA (**a**) and RTA (**b**). Annealing was performed under a N_2_ or O_2_ atmosphere as indicated in the figures.

As shown in Figure 4a,b, after FLA or RTA, the implanted PZO recrystallizes. The broad peaks caused by disordered grains shown in as-implanted samples disappear, and the peak located between 570 cm^−1^ and 585 cm^−1^ occurs in the Raman spectra, which could be assigned to the A_1_^(LO)^ out-of-plane vibration phonon mode or the E_1_^(LO)^ phonon mode due to the in-plane vibration within the wurtzite ZnO lattice [50]. In the case of FLA performed in N_2_ atmosphere, the samples show the phonon mode located close to 572 cm^−1^, while after annealing in O_2_, the main phonon mode is closer to 582 cm^−1^. In the case of RTA-treated PZO, the new peaks are located at 582 cm^−1^. Since the A_1_ phonon mode is usually observed at a lower frequency than E_1_ (shifted to a lower wavenumber by about 10 cm^−1^), and in samples with defects causing the symmetry breaking, this suggests that FLA in nitrogen promotes the formation of oxygen vacancies while annealing in oxygen more efficiently neutralizes the point defects like oxygen vacancies and Zn interstitials. Moreover, P-doped ZnO grown at 130 °C exhibits an additional phonon mode at about 275 cm^−1^, which was not found within samples grown at 200 °C. The active Raman phonon mode at 275 cm^−1^ was found and reported first in nitrogen (N)-doped ZnO, and the peak intensity had a linear relationship with N concentration. It was interpreted as a N-related mode [51]. Massazza et al. [3] identified this peak with the vibration of an interstitial Zn atom bound to a substitutional nitrogen atom (Zn_i_-N_O_). However, this conclusion was ruled out soon because these vibrational modes were also found in ZnO doped with other elements, such as Al, Sb, Fe, and Ga [52]. As these elements come from different groups and periods and have different atomic structures, the phonon mode located at about 275 cm^−1^ is considered to be caused by intrinsic defects in the host lattice, which either become activated as vibrating complexes or their concentration increases upon dopant incorporation [52]. Later, this peak was found also in undoped pulse-laser-deposited ZnO without O_2_ plasma treatment and ascribed to vibrations within Zn clusters [53]. Until now, there has been no consensus on the origin of this peak in the literature. In our work, this phonon mode is present in samples grown at 130 °C, which suggests that it can be due to some residual defects.

**Figure 5:**
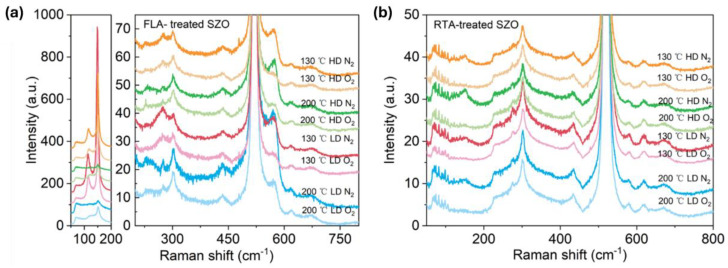
Raman spectra of Sb-implanted samples grown at different temperatures and annealed either in a N_2_ or in a O_2_ atmosphere using FLA treatment (**a**) or RTA (**b**). The low-frequency part of the Raman spectra from (**a**) is shown separately at a different scale due to very intense peaks.

**Figure 6:**
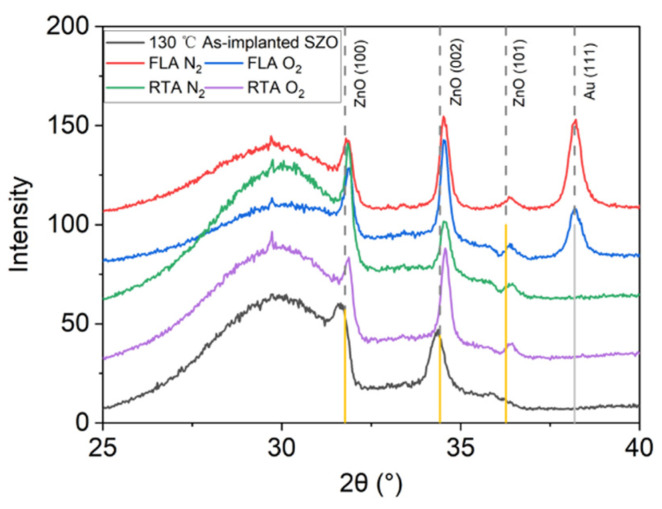
XRD patterns of Sb-implanted samples grown at 130 °C and annealed either in a N_2_ or in a O_2_ atmosphere using FLA or RTA treatment.

Figure 5 presents the Raman spectra recorded from the samples implanted with Sb and annealed by FLA or RTA in different atmospheres. As we can see from Figure 5a, after FLA sharp peaks appeared at about 115 cm^−1^ and 150 cm^−1^ indicating the presence of metallic Sb clusters [54] and a new peak at about 572 cm^−1^ caused by the out-of-plane vibration, namely the A_1_ phonon mode. The RTA-treated SZO films feature new peaks at 582 cm^−1^ due to the E_1_ in-plane vibration mode. The annealing atmosphere affects only the peak intensities, while the peak positions remain unchanged. Samples with the strongest phonon modes related to the Sb clusters were measured by XRD, also shown in Figure 6. Unfortunately, we could not see the characteristic diffraction peak that would be assigned to crystalline Sb inclusions. The XRD measurements confirm the disorder formation in the as-implanted samples but without full amorphization. Moreover, the average crystal size calculated from the width of the ZnO 002 reflection using the Scherrer formula increases from about 20 nm in the as-implanted stage to about 30 nm after RTA in the N_2_ atmosphere. The peak at about 38° is due to the gold contacts.

### 3.4. PL Spectroscopy

**Figure 7:**
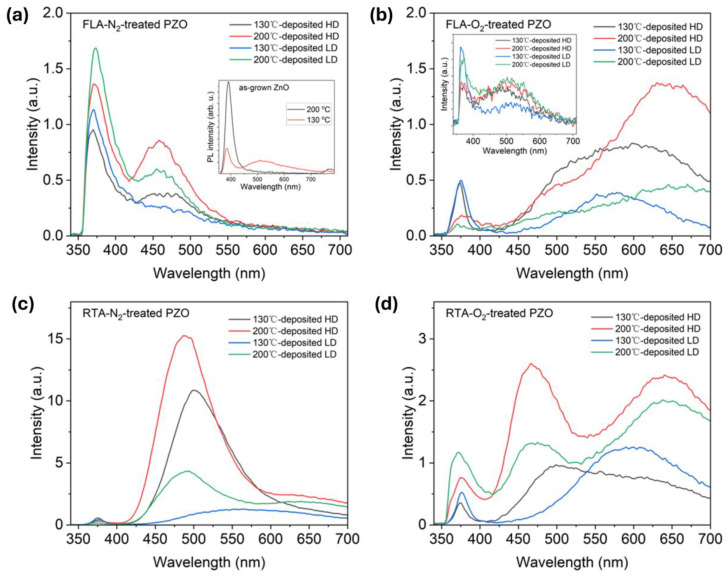
Room temperature PL spectra of (**a**) FLA-N_2_-treated, (**b**) FLA-O_2_-treated, (**c**) RTA-N_2_-treated, and (**d**) RTA-O_2_-treated PZO grown at 130 °C and 200 °C, and then implanted with different dopant doses, respectively. The insets in (**a**,**b**) show the PL spectra obtained from as-grown and as-implanted ZnO, respectively.

To further understand the crystal dynamics under-investigated fabrication parameters, PL spectroscopy was applied to measure the optical properties of implanted and annealed ZnO films. At room temperature, the as-implanted samples show only a very weak near-bandgap emission (NBE) at about 370 nm and green defect emission at about 500 nm. The PL from as-deposited samples shows strong NBE for samples grown at 200 °C while the PL spectrum from samples grown at 130 °C is composed of the NBE and defect-related emissions (see inset Figure 7a). Overall, the PL emission from the as-implanted sample is very weak. Nevertheless, the presence of NBE in as-implanted samples confirms the existence of short-range order in the crystal structure, e.g., nanocrystalline ZnO that is not fully destroyed by ion implantation (see the inset Figure 7b). The green emission can originate from an optical transition within zinc vacancies (V_Zn_) [55,56] or oxygen vacancies (V_O_) [57,58].

After thermal treatment, P-implanted layers are recrystallized. Figure 7 shows the evolution of the RT PL as a function of annealing and implantation parameters including samples grown at different temperatures. After FLA in nitrogen, the P-implanted samples exhibit mainly the NBE emission at about 370 nm and a blue-green emission at about 460 nm due to Zn_i_ [59]. The emission from the PZO sample annealed under the O_2_ atmosphere is dominated by radiative recombination within defects, i.e., broad peaks comprising green emission at about 500 nm assigned with zinc vacancies (V_Zn_) or oxygen vacancies (V_O_) and red emission (~620 nm) attributed to oxygen interstitials (O_i_) [60]. Meanwhile, the NBE emission after annealing in oxygen is stronger for samples implanted with a lower fluence. Unlike PZO treated by FLA, RTA treatment promotes defect formation inside thin films (see Figure 7c,d). The PL spectrum after RTA in nitrogen is dominated by the green emission, most probably due to the radiative recombination at oxygen vacancies. During high-temperature annealing, oxygen can diffuse out, which increases the concentration of V_O_. Simultaneously, the NBE is very weak in all investigated samples. The PL spectra obtained after RTA in oxygen consist of three main emissions: NBE at about 375 nm, green emission from V_Zn,_ and red emission from O_i_.

**Figure 8:**
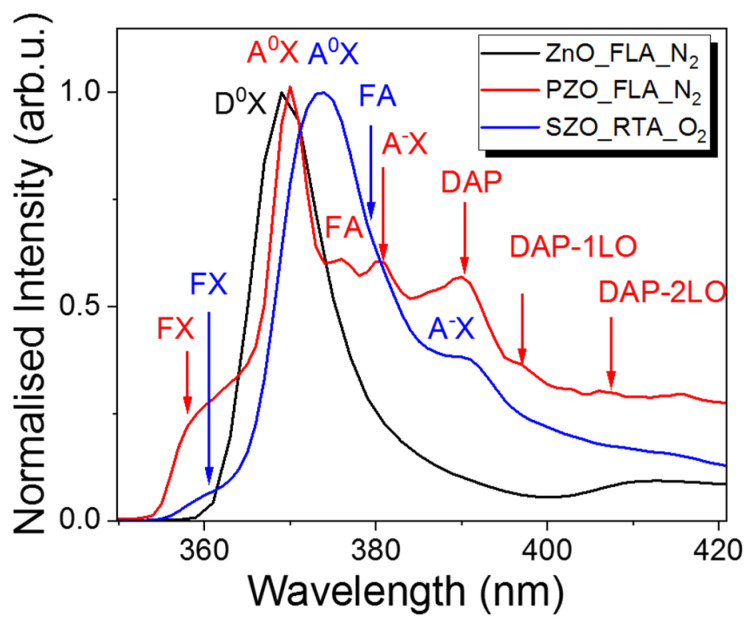
Low-temperature PL spectra taken from un-doped ZnO and P- or Sb-doped samples. All samples were grown at 130 °C and subsequently annealed.

The incorporation of dopants into ZnO can be verified by low-temperature PL investigations. Figure 8 shows the normalized PL spectra taken at 15 K from undoped ZnO and P or Sb implanted samples after annealing. We have chosen the P-doped sample after FLA in nitrogen ambient since this one has the highest hole concentration and an Sb-doped sample after RTA in O_2_ due to the oxidation of Zn interstitial atoms during annealing. Zn interstitials are responsible for n-type doping and blue emission in ZnO [16]. The flash annealed and undoped sample shows a single peak at 369.0 nm (3.345 eV), which can be assigned to neutral donor–bound excitons (D^0^X) [61]. After P-doping, the main peak is slightly red-shifted by 2 nm with a higher energy shoulder peak at about 359.1 nm (3.443 eV). The high energy emission peak can be due to free exciton transition denoted by FX while the main peak at about 370.1 nm (3.339 eV) is due to a neutral acceptor–bound exciton transition (A^0^X). On the lower energy part, the PL spectrum from P-doped ZnO exhibits more optical transitions. The first shoulder peak of A^0^X emission observed at about 375.8 nm (3.289 eV) we designed to free electron to acceptor (FA).

The peak at about 380.7 nm (3.246 eV) is due to the optical transition of the ionized acceptor–exciton transition (A-X). Moreover, the P-doped sample shows donor-to-acceptor pair optical transitions (DAP) and their longitudinal optical phonon replicas (DAP-1LO and DAP-2LO) [62]. The low-temperature PL emission from an Sb-doped sample and annealed in oxygen by the RTA process shows the main peak due to a neutral acceptor–bound exciton transition at about 373.5 nm (3.292 eV) and the A-X transition appears at 390.8 nm (3.163 eV). The free exciton transition in SZO is observed at 360.9 nm (3.425 eV). The red shift of the PL emission after Sb doping is expected since the Sb in ZnO is assumed to be a deep acceptor with an ionization energy of about 160 meV [63].

## 4. Conclusions

In this study, we have shown that even polycrystalline ZnO can be converted from n-type to p-type semiconductors. The p-type doping of ZnO films was realized by P ion implantation followed by FLA for 23 ms or a conventional RTA treatment. Samples annealed by FLA show the hole concentration of 1.9 × 10^18^ cm^−3^ which is the highest hole concentration obtained for P-implanted ZnO. In contrast, Sb-doped samples show only n-type conductivity independent of the post-implantation annealing process and formation of metallic Sb clusters as revealed by Raman spectroscopy. The annealing in nitrogen ambient provides stronger NBE. Annealing in oxygen promotes oxygen diffusion into the film and thus the red PL emission. Moreover, we have shown that the annealing atmosphere strongly influences the optoelectronic properties of ion-implanted ZnO. Annealing in nitrogen promotes the formation of oxygen and zinc vacancies while annealing in oxygen causes the oxygen in-diffusion and formation of oxygen interstitials. Finally, we have proven that the two strongly non-equilibrium processes like ion implantation and flash lamp annealing can be used to fabricate p-type ZnO even in its polycrystalline form. To finally verify the universal behavior of non-equilibrium processing for p-type doping of ZnO, different growing techniques including PLD or magnetron sputtering must be tested. In principle, presented controllable p-type doping of wide bandgap oxides is a milestone for their broad application in future optoelectronics.

## Figures and Tables

**Table 1:** P and Sb ion implantation parameters for ZnO thin films.

Implanted Element	Low Implantation Dose			High Implantation Dose		
	Energy (keV)	Fluence (cm^−2^)	Dopant Concentration (cm^−3^)	Energy (keV)	Fluence (cm^−2^)	Dopant Concentration (cm^−3^)
Phosphorus (P)	90	7.5 × 10^15^	8 × 10^20^	90	1.5 × 10^16^	1.6 × 10^21^
	30	1.5 × 10^15^		30	3 × 10^15^	
Antimony (Sb)	300	6 × 10^15^	8 × 10^20^	300	1.2 × 10^16^	1.6 × 10^21^
	100	1.7 × 10^15^		100	3.4 × 10^15^	

**Table 2:** Carrier concentration and carrier mobility of ion-implanted doped ZnO samples.

Growth and Implantation Conditions	Carrier Concentration (cm^−3^)/Mobility (cm^2^/Vs)				
	Not Annealed	FLA, N_2_, 23 ms	FLA, O_2_, 23 ms	RTA, N_2_, 1 min	RTA, O_2_, 1 min
PZO 130 °C LD	−4.70 × 10^20^	1.91 × 10^18^/4.65	−3.05 × 10^19^	1.15 × 10^16^/10.08	2.21 × 10^16^/4.90
PZO 130 °C HD	−7.13 × 10^20^	−1.22 × 10^18^	6.98 × 10^17^/4.65	1.87 × 10^16^/12.71	1.55 × 10^14^/-
PZO 200 °C LD	−5.06 × 10^20^	2.84 × 10^17^/6.72	1.87 × 10^18^/2.81	6.00 × 10^14^/-	1.21 × 10^16^/6.34
PZO 200 °C HD	−7.06 × 10^20^	−3.41 × 10^20^	−2.95 × 10^17^	6.36 × 10^14^/-	3.38 × 10^16^/8.39
SZO 130 °C LD	−4.53 × 10^19^	−5.57 × 10^19^	−3.58 × 10^19^	uncertain	uncertain
SZO 130 °C HD	−2.82 × 10^19^	−5.12 × 10^19^	−6.53 × 10^19^	uncertain	uncertain
SZO 200 °C LD	−5.27 × 10^19^	−4.42 × 10^19^	−2.55 × 10^19^	uncertain	uncertain
SZO 200 °C HD	−5.85 × 10^19^	−5.11 × 10^19^	−3.58 × 10^19^	uncertain	uncertain

## Data Availability

The data sets generated and/or analyzed during the current study are available from the corresponding author upon reasonable request.
